# Impact of thresholding on the consistency and sensitivity of diffusion MRI‐based brain networks in patients with cerebral small vessel disease

**DOI:** 10.1002/brb3.2523

**Published:** 2022-04-12

**Authors:** Bruno M. De Brito Robalo, Naomi Vlegels, Alexander Leemans, Yael D. Reijmer, Geert Jan Biessels

**Affiliations:** ^1^ Department of Neurology and Neurosurgery UMC Utrecht Brain Center University Medical Center Utrecht Utrecht The Netherlands; ^2^ PROVIDI Lab Image Sciences Institute University Medical Center Utrecht Utrecht University Utrecht The Netherlands

**Keywords:** cerebral small vessel disease, diffusion tensor imaging, network density, network reproducibility, network thresholding

## Abstract

**Introduction:**

Thresholding of low‐weight connections of diffusion MRI‐based brain networks has been proposed to remove false‐positive connections. It has been previously established that this yields more reproducible scan–rescan network architecture in healthy subjects. In patients with brain disease, network measures are applied to assess inter‐individual variation and changes over time. Our aim was to investigate whether thresholding also achieves improved consistency in network architecture in patients, while maintaining sensitivity to disease effects for these applications.

**Methods:**

We applied fixed‐density and absolute thresholding on brain networks in patients with cerebral small vessel disease (SVD, *n* = 86; ≈24 months follow‐up), as a clinically relevant exemplar condition. In parallel, we applied the same methods in healthy young subjects (*n* = 44; scan–rescan interval ≈4 months) as a frame of reference. Consistency of network architecture was assessed with dice similarity of edges and intraclass correlation coefficient (ICC) of edge‐weights and hub‐scores. Sensitivity to disease effects in patients was assessed by evaluating interindividual variation, changes over time, and differences between those with high and low white matter hyperintensity burden, using correlation analyses and mixed ANOVA.

**Results:**

Compared to unthresholded networks, both thresholding methods generated more consistent architecture over time in patients (unthresholded: dice = .70; ICC: .70–.78; thresholded: dice = .77; ICC: .73–.83). However, absolute thresholding created fragmented nodes. Similar observations were made in the reference group. Regarding sensitivity to disease effects in patients, fixed‐density thresholds that were optimal in terms of consistency (densities: .10–.30) preserved interindividual variation in global efficiency and node strength as well as the sensitivity to detect effects of time and group. Absolute thresholding produced larger fluctuations of interindividual variation.

**Conclusions:**

Our results indicate that thresholding of low‐weight connections, particularly when using fixed‐density thresholding, results in more consistent network architecture in patients with longer rescan intervals, while preserving sensitivity to disease effects.

## Graphical abstract

In this study, we investigated whether thresholding methods that have been shown to improve scan–rescan reproducibility of diffusion‐based brain networks in healthy young subjects are also applicable to datasets of elderly patients with brain pathology, scanned over much longer time frames. We examined whether thresholding achieves improved consistency in network architecture, while maintaining sensitivity to biological effects in patients with cerebral small vessel disease.

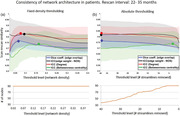

## 1 INTRODUCTION

Diffusion‐weighted imaging (DWI) and fiber tractography enable us to map cerebral white matter pathways and reconstruct large‐scale brain networks (Jeurissen et al., 2017). Subsequently, graph theory can be applied to quantify the properties of such networks (Hagmann et al., 2007; Sporns et al., 2005). This framework has been widely used to investigate not only normal brain development but also a variety of neurological and psychiatric disorders (Fornito et al., 2013; Tijms et al., 2013).

A major challenge in structural network analysis is the limited reproducibility of networks obtained with diffusion MRI, due to the presence of false‐negative and false‐positive connections (Buchanan et al., 2014; Zalesky et al., 2016). Essentially, false negatives represent white matter connections that are undetected by the tractography algorithm. By contrast, false positives are edges in the reconstructed network that do not represent true white matter connections. These errors can result from numerous processing steps (Sotiropoulos & Zalesky, 2017), particularly the choice of parcellation scheme (Zalesky et al., 2010), tractography algorithm (Sarwar et al., 2018), and weighting strategy (Dimitriadis et al., 2017).

The most common solution to reduce false positives is to employ weight‐based thresholding by removing so‐called “weak” connections. In diffusion‐based brain networks, weak connections are usually defined as having a low number of streamlines (NOS). Many network studies have used thresholding strategies such as absolute thresholding (Garrison et al., 2015; Nicols et al., 2016), where a uniform threshold is applied to remove all connections below a certain edge‐weight (e.g., below five streamlines). Another popular method is fixed‐density thresholding (Rubinov & Sporns, 2010; van den Heuvel et al., 2017), where a relative threshold derived from each individual's data is applied to remove the weakest connections, such that an equal network density is achieved across subjects. Recent scan–rescan studies have investigated the impact of thresholding on the reproducibility of structural brain networks in healthy subjects (Buchanan et al., 2020; Messaritaki et al., 2019; Sarwar et al., 2018; Tsai, 2018; Welton et al., 2015). Their results suggest that applying thresholds to remove false positives can improve network similarity between scan and rescan, not only in terms of graph metrics but also by replicating the same network architecture (e.g., the same set of edges and edge‐weights).

Previous scan–rescan studies that tested reproducibility typically focused on datasets of healthy subjects, with state‐of‐the art MRI sequences, high imaging quality, and short rescan intervals (Van Essen et al., 2013). Thresholding methods may also be of value to increase consistency in network architecture in datasets of patient populations. In this setting, diffusion‐based network studies are primarily used to study disease effects, cross‐sectionally and over time. Before application in such clinical studies, it is essential to understand if thresholding indeed also produces more consistent network architecture in scans from patients, acquired in a clinical setting, containing various degrees of pathology, and across longer rescan intervals where further pathology has likely occurred. It is also important to determine if gain in network architecture consistency in this setting does not come at the cost of reduced sensitivity to detect disease effects, reflected in interindividual variation in diffusion metrics, and disease‐related network changes over time.

In this study, we therefore investigated whether thresholding methods that were previously shown to improve reproducibility in repeated scans of healthy young subjects also generate more consistent network architectures (e.g., the same set edges, edge‐weighs, and hubs‐scores) in patients who were scanned over longer time periods. To this end, we used longitudinal data of patients with cerebral small vessel disease (SVD), a condition known to affect cerebral white matter integrity that is often investigated with network analysis (Lawrence et al., 2018; Reijmer et al., 2013). In addition, we evaluated in these patients how thresholding affects sensitivity to disease effects, which was defined as (1) interindividual variation in network measures often examined in SVD (e.g., global efficiency and node strength) and (2) differences in global efficient and node strength between patients with low versus high SVD disease burden. We focused on two thresholding methods commonly applied in brain network studies: absolute thresholding and fixed‐density thresholding, both of which remove low‐weight connections and allow analysis of scan–rescan reproducibility on an individual patient level. As a frame of reference, we processed a dataset of healthy controls with the same methodology.

## 2 MATERIALS AND METHODS

### 2.1 Dataset 1—Memory clinic patients with SVD

We included 228 patients from the Parelsnoer longitudinal study (Aalten et al., 2014). Patients who were referred to the memory‐clinic of the UMC Utrecht for evaluation of cognitive problems, with a clinical dementia rating scale (CDR) (Morris, 1993) score of 0, .5, or 1, and a Mini Mental State Examination (MMSE) (Folstein et al., 1975) of 20 or higher were eligible. Exclusion criteria were: normal pressure hydrocephalus, Morbus Huntington, recent transient ischemic attack (TIA) or cerebrovascular accident (CVA) (<2 years), TIA/CVA followed by cognitive decline (within 3 months), history of major psychiatric disease or brain disease other than neurodegeneration or vascular disease, causing cognitive decline (e.g., brain tumor, epilepsy). Patients were eligible for the current analysis if they had a structural MRI and DTI scan at baseline and after ≈2‐year follow‐up visit date (*N* = 90). We additionally excluded three patients with cognitive complaints due to a diagnosis other than SVD or Alzheimer's disease (AD) to obtain a more homogenous study sample and one patient who was an extreme outlier in the network analysis. Thus, the total number of subjects included in the analysis was 86 (59% male). The follow‐up time ranged between 22 and 35 months (mean ± SD: 27 ± 3 months) and age of the patients varied between 56 and 86 years (mean ± SD: 73 ± 7 years). MRI data were acquired on a 3 tesla Philips scanner (Achieva, Philips, Best, the Netherlands) using a standardized clinical protocol that included a 3D T1‐weighted image and a diffusion weighted sequence. T1‐weighted scans were acquired with a voxel size of 1 mm^3^. DWI scans had an isotropic acquisition voxel size of 2.50 mm^3^, 45 diffusion‐sensitizing gradients with a b‐value of 1200 s/mm^2^, and 1 b = 0 s/mm^2^. Fluid‐attenuated inversion recovery (FLAIR; TR/TE/inversion time: 11,000/125/2800 ms) were also obtained.

The study was approved by the institutional review board of the UMC Utrecht, and all participants provided written informed consent prior to any research procedure.

### 2.2 Dataset 2 – Reference data of healthy young adults

As a frame of reference, we included a second dataset with repeated scans from healthy young adults from the Human Connectome Project (HCP, Van Essen et al., 2013). Previous studies have already tested the effects of thresholding using on this dataset but since network reconstruction pipelines always differ slightly across studies. We included these controls in our study to have a high‐quality reference, reconstructed with the exact same software packages, and tractography algorithm as our patient data. We selected 44 healthy participants (32% male) with scan–rescan DWI and T1‐weighted images. The rescan interval ranged between 1.5 and 11 months (mean ± SD: 4.7 ± 2 months) and the age of the participants varied between 22 and 35 years old. MRI was acquired on a Siemens Skyra 3 tesla scanner (Siemens, Erlangen, Germany). T1‐weighted images had an isotropic voxel size of 1.25 mm^3^. The multi‐shell DWI were acquired with an isotropic voxel size of 1.25 mm^3^ and three diffusion weightings (b‐values: 1000, 2000, and 3000 s/mm^2^). For each b‐value, 90 diffusion‐sensitizing gradients directions were measured. Additionally, 18 images with no diffusion weighting (b‐values = 0 s/mm^2^) were obtained. Here, we selected only a single shell (b‐value 1000 s/mm^2^), since it was more comparable to the patient dataset described above.

### 2.3 Diffusion processing and fiber tractography

All DWI scans were processed using ExploreDTI version 4.8.6 (Leemans et al., 2009) running on MATLAB R2018a (MATLAB and Statistics Toolbox Release 2018a, The MathWorks, Inc., Natick, MA, USA). Data were corrected for signal drift (Vos et al., 2017), eddy currents, subject motion with rotation of the B‐matrix (Leemans & Jones, 2009), and susceptibility distortions (Veraart et al., 2013). The DWI volumes were nonlinearly registered to the T1 images prior to estimation of the diffusion tensors. Diffusion tensors were estimated using a robust method to account for outliers (Tax et al., 2015), and fiber tracts were reconstructed using deterministic fiber tractography. Seed points were distributed uniformly throughout the whole brain with 2 mm isotropic resolution. The streamlines were propagated using integration over fiber orientation distributions (FOD), with a step size of 1 mm. The orientation distributions were inferred using constrained spherical deconvolution (CSD) with a maximum harmonic order (*l‐max*) of 6 (Jeurissen et al., 2011). Fiber tracking was terminated when streamlines entered a voxel with FOD < .1, or when the deflection angle between two successive 1 mm steps was > 45°. When tractography was concluded, streamlines with a length outside of the range between 10 and 500 mm were excluded.

### 2.4 Network definition

**FIGURE 1:**
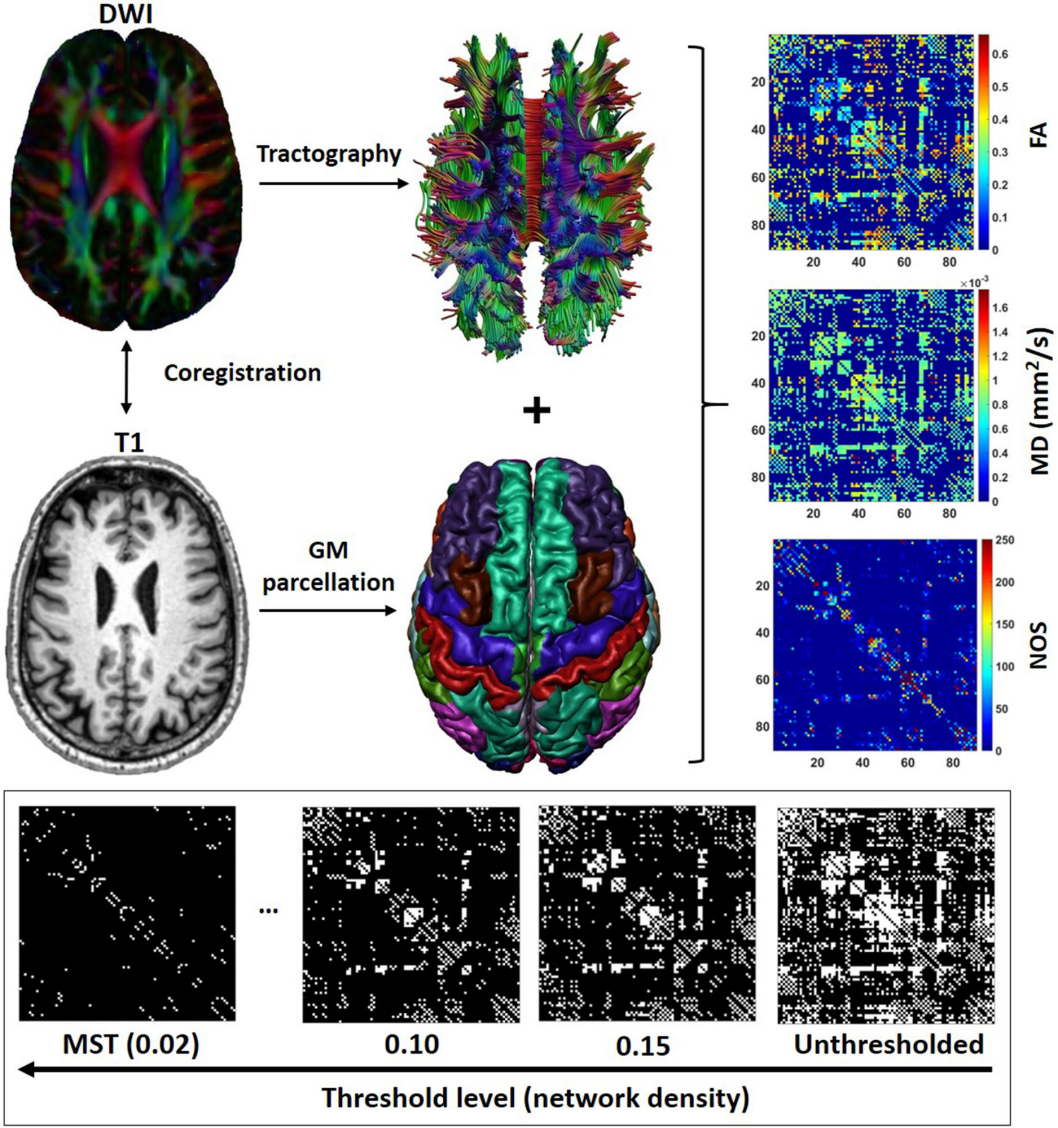
Network definition and thresholding. First, the DWI images were reregistered to the T1 and corrected for subject motion and artefacts. This was followed by fiber tractography and gray matter (GM) parcellation. The tractography image and segmented brain regions were combined to obtain 90×90 connectivity matrices weighted by fractional anisotropy (FA), mean diffusivity (MD), and number of streamlines (NOS). The NOS matrix was used for thresholding. The thresholded networks obtained at each threshold level were subsequently weighted by FA and MD, and used in further analysis

Figure 1 illustrates the processing steps for network definition. The T1‐weighted scans were preprocessed using the FMRIB Software Library (FSL v5.0, Smith et al., 2004) and the Computational Anatomy Toolbox (CAT12, http://dbm.neuro.uni‐jena.de/cat). First, each subject's T1 image was skull‐stripped using FSL BET (Smith et al., 2004). Next, the gray matter volume was parcellated into 90 cortical and subcortical regions of interest (ROIs) using the automated anatomical labeling (AAL) atlas (Tzourio‐Mazoyer et al., 2002). The parcellations were performed in the native T1 space, with the AAL template being nonlinearly registered to each subjects T1 image. The parcellated regions and the tractography data were combined to reconstruct the whole‐brain network. Each ROI represented a node in the network, and two nodes were considered to be connected when they contained the end‐points of at least one streamline, resulting in a 90 × 90 binary connectivity matrices. We also computed three weighted three matrices, where the edges were weighted by the number of streamlines (NOS) connecting the two nodes, the mean diffusivity (MD), and the fractional anisotropy (FA).

### 2.5 Network thresholding

Thresholding is frequently applied after network reconstruction, aiming to reduce the number of false‐positive connections. In this work, we employed the two most common weight‐based thresholding strategies: fixed‐density thresholding and absolute thresholding. The fixed‐density approach involved removing the edges with the lowest NOS until an equal density was achieved for all subjects. Network density is defined as the proportion of actual connections in the network, relative to all possible connections. For example, when a network has a density of .15, it means that 15% of all possible connections were detected in that network. To ensure that the networks did not become disconnected after thresholding we incorporated the minimal spanning tree (MST), an acyclic subgraph that connects all *N* nodes in the network (Tewarie et al., 2015). The MST is computed at the beginning of the thresholding step by selecting only edges with the highest NOS unless an edge forms a cycle. When all nodes are connected, the MST has *N* − 1 connections and a density of 2/*N* (≈.02), with *N* being the number of nodes (*N* = 90 nodes in our case). Using the MST as a starting point, fixed‐density thresholding is applied by adding more edges to the network (from strongest to weakest weights) until a certain density is achieved. We varied the density level between the density of the MST (.02) and the mean density of all unthresholded networks (density = .40) in steps of .01. Note that the stronger the threshold level, the lower the network density. The absolute thresholding approach involved removing all edges with a weight below an absolute number of streamlines. We varied the absolute threshold level between 1 and 40 streamlines in steps of 1. In this case, the stronger the threshold, the higher the number of streamlines removed. For example, a threshold of 20 streamlines means that that have fewer than 20 streamlines are removed from the network. This thresholding method does not ensure that nodes will not become disconnected or that networks of different subjects will have the same density after thresholding.

### 2.6 WMH volume segmentation

WMH hyperintensity volumes were segmented from the FLAIR images using and automated pipeline, kNN‐TTP (Steenwijk et al., 2013).

### 2.7 Consistency of network architecture

To examine the consistency of network architecture between scan and rescan, we focused on characteristics that represent the building blocks of structural networks, such as edges detected, edge‐weight distribution, and hub‐scores (node degree and betweenness centrality).

#### 2.7.1 Similarity in edges detected

The most direct manner of measuring similarity between networks of scan and rescan is to overlap the edges detected at both time points. Using the binary connectivity matrix, we computed the dice similarity coefficient between edges detected at scan and rescan:

$$dice=\frac{2\;\left|scan\cap rescan\right|}{\left|scan\right|+\left|rescan\right|}$$

Here, |scan∩rescan| represent edges in common between the two scans, whereas |*scan*| and |*rescan*| represent unique edges of scan and rescan, respectively. The dice coefficient ranges from 0 to 1, with 0 indicating no overlap and 1 representing a complete overlap between the two sets of edges.

#### 2.7.2 Similarity in edge‐weight

We also evaluated whether the edges detected in both scans have similar weights, namely the number of streamlines (i.e., the weight used to determine which edges should be retained or removed). Thus, we first computed edges in common between scan and rescan and calculated the agreement in edge‐weight using the intraclass correlation coefficient (ICC) (Shrout & Fleiss, 1979). The ICC was originally created to assess the reliability of multiple raters measuring the same variable, but it is also often utilized in network studies to assess the consistency of graph measure over multiple sessions (Andreotti et al., 2014; Buchanan et al., 2014; Messaritaki et al., 2019):

$$\mathrm{ICC}\;=\;\frac{MS_{b}-MS_{w}}{MS_{b}+\left(k-1\right)MS_{w}}$$

where $MS_{b}$ is the between‐subject variance, $MS_{w}$ represents within‐subject variance, and k is the number of repeated measurements. ICC values range between 0 and 1 and are typically interpreted as poor (<.40), fair (.40−.59), good (.60−.74), and excellent (>.75) (Cicchetti, 1994; Wang et al., 2019; Yuan et al., 2019).

#### 2.7.3 Similarity in hub score

Another relevant feature of network architecture is the location of hub nodes. Hubs are topologically central regions and are positioned to make strong contributions for global network function (van den Heuvel & Sporns, 2013). The two most common graph metrics used to define node importance and to identify hubs are node degree and node betweenness centrality. Degree refers to the number of connections that link one node to adjacent nodes. Betweenness centrality is defined as the fraction of all shortest paths in the network that pass through a given node (Bullmore & Sporns, 2009). These nodes with high “hub‐score” (i.e., high degree and/or betweenness centrality) contribute to an efficient communication between distant brain regions. We compared the similarity in hub scores of all nodes between scan and rescan by computing the ICC of betweenness centrality and ICC of degree.

### 2.8 Interindividual variation and sensitivity to changes over time in patients

Here, we examined how thresholding affects the natural interindividual variation in the data, necessary to test associations with external variables and perform group comparisons (Bagarinao et al., 2019). We focused on metrics such as global efficiency and FA‐ and MD‐weighted node strength. Global efficiency was defined as the inverse of the average shortest path length and quantifies how efficiently information is exchanged over the network (Rubinov & Sporns, 2010). Node strength was defined as the average FA or MD of all edges connected to a node. Clearly, disturbances of these network metrics are not specific to only SVD. Moreover, a range of other metrics exist. We choose these particular measures because they are known to be affected by SVD (Lawrence et al., 2014; Reijmer et al., 2013). Moreover, these metrics (i.e., lower global efficiency and FA, and a higher MD) are known to be related to disease burden and progression over time. Patients with larger WMH volumes show lower global efficiency and FA, and a higher MD, and as the disease progresses over time, global efficiency, FA and MD are expected to decline further (Tuladhar et al., 2020). Therefore, we also tested among the patients how thresholding affected the effect size of the difference in diffusion metrics between high and low disease burden and sensitivity to detect changes over time. The paragraphs below describe how we evaluated the interindividual variation of these metrics and sensitivity to detect time and group effects for different threshold levels.

#### 2.8.1 Assessing interindividual variation

**FIGURE 2:**
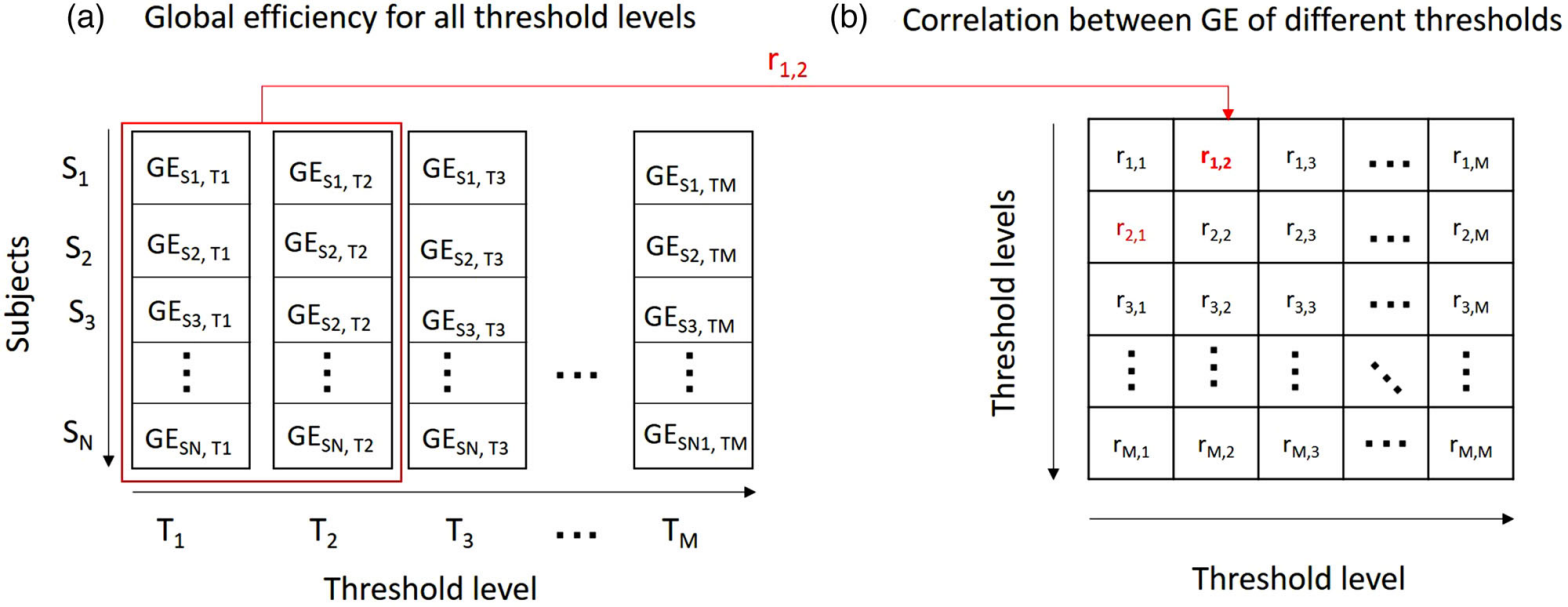
Correlation matrix of global efficiency values of different thresholds. (a) First, for each threshold level (T), z‐scores of global efficiencies (GE) were calculated for all subjects (S). Then, to examine whether the inter‐individual variation changes between threshold levels (e.g., level 1 and 2), we calculated the Pearson correlation coefficient between global efficiency of those threshold levels (r1,2), resulting correlation matrix containing correlations between all pairs of threshold levels. (b) Correlation coefficients were calculated for all combination of thresholds, resulting in a correlation matrix. Note that this matrix is symmetric since *r*1,2 = *r*2,1

For each threshold level, global efficiency and node strength were calculated and transformed to z‐scores. Follow‐up z‐scores were calculated using the mean and standard deviation of baseline. To assess whether thresholding affects interindividual variation, we computed a correlation matrix containing Pearson's correlation coefficients between z‐scores of different threshold levels. Figure 2 shows an example of how this correlation matrix was calculated for global efficiency (GE). First, global efficiency z‐scores were calculated for each subject (S) and for each threshold level (T). Then, z‐scores of one threshold level were correlated with z‐scores of another threshold level, yielding a correlation coefficient (*r*). The strength of this correlation indicates whether the interindividual variation in global efficiency is preserved between threshold levels. In other words, if baseline scores change between threshold levels, this results in low correlation coefficients. We calculated *r* between all combinations thresholds to fill the correlation matrix. In this manner, it is possible to observe which threshold levels generate substantially different global efficiency values. We also computed the same correlation matrix for rate of the change in global efficiency over time (i.e., baseline minus follow‐up), to understand whether thresholding affects the estimated individual rate of change over time. Essentially, for a hypothetical subject with a baseline global efficiency of 1.5×standard deviation (SD) relative to the group mean, if the interindividual variation is not affected, the same score should be obtained for that subject when a different threshold is used. Similarly, if the global efficiency of that subject declined −.5 SD from baseline to follow‐up, the same rate of decline should be observed at different thresholds. The range of thresholds that shows the highest *r* values thus represent the thresholds were sensitivity to interindividual variation is optimal. Differences in follow‐up time between subjects were not adjusted in the analyses, because such differences only further contribute to interindividual variation and our intention was to assess that variation.

#### 2.8.2 Assessing changes over time

To examine whether thresholding affects the sensitivity to detect network changes over time in patients, we used mixed ANOVA to compare baseline versus follow‐up global efficiency and node strength. Patients were stratified into two groups using a median split of WMH volume. For this analysis, 13 patients were excluded due to lack of FLAIR images for the segmentation of WMH, resulting in 73 patients. We evaluated whether the sensitivity to detect an effect of time (within‐subject factor), effect of group (between‐subject factor), and interaction time × group is preserved across thresholds.

## 3 RESULTS

### 3.1 Consistency of network architecture

**FIGURE 3:**
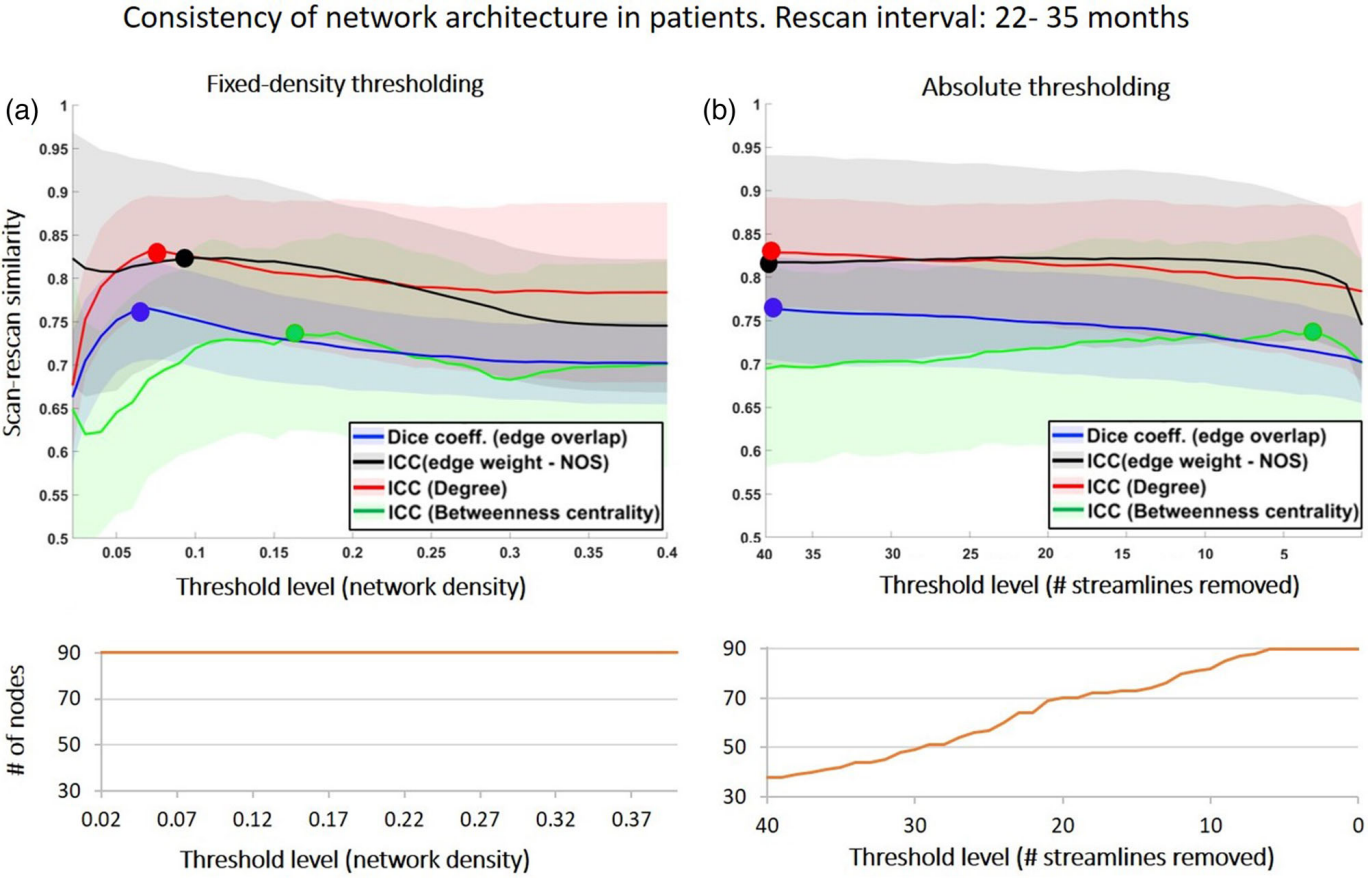
Consistency of network architecture between scan and rescan in patients (rescan interval = 22–35 months). In each plot, the x‐axis represents the threshold level, with the strength of thresholding increasing from right to left. Note that for the fixed‐density approach the stronger the threshold the lower the density, whereas for the absolute approach, the stronger the threshold, the higher the number of streamlines removed. The curve in orange under each plot shows the number of nodes that remain in the network after thresholding. The initial number of nodes for the unthresholded network was *N* = 90 nodes. Blue: dice similarity of edges, black: ICC of edge‐weights; red: ICC of degree; green: ICC of betweenness centrality. The markers highlight maximum value in each curve, and the shaded areas represent the standard deviation

Figure 3 shows the effect of thresholding on the consistency of network architecture in patients. In each panel, the first plot contains similarity scores for different characteristics of the network architecture, and the second plot shows the number of nodes that remain in the network at each threshold level. Quantitate values are shown in Table S1. Results with the reference dataset of controls are shown in the Supporting Information (Figure S1 and Table S2).

#### 3.1.1 Similarity in edges detected

In the patients, the dice similarity of edges was .70 before thresholding. When fixed density thresholding was applied, this score increased with stronger thresholds (i.e., with lower densities), reaching a maximum of .76 (at a density = .08, *p* < .001 compared to unthresholded, Figure 3a, blue line, Table S1). For densities lower than .08, dice scores decreased sharply to .67 (density = .02). As expected, the number of nodes (*N* = 90) in the network did not change with fixed‐density thresholding. Regarding absolute thresholding, the dice score also increased with stronger thresholds (i.e., with larger number of streamlines removed), from .70 (unthresholded) to a maximum of .76 (# streamlines removed = 40, *p* < .001, Figure 3b, blue, Table S1). With absolute thresholding, the maximum dice score was produced by the strongest threshold. However, at this threshold level, only 38 of the initial 90 nodes remained in the network. Overall, these results indicate that compared to not applying any threshold whatsoever, thresholding generates a more similar set of edges between scan and rescan.

In controls, thresholding had a similar effect on dice similarity, albeit with higher scores than in patients as expected, because of higher quality scans and absence of pathology (Figure S1A and B, blue, Table S2).

#### 3.1.2 Similarity in edge‐weight

The ICC of edge‐weight in patients was .71 for unthresholded networks (Figure 3a, black). After fixed‐density thresholding, ICC scores increased with stronger thresholds to a maximum of .75 (density = .12, *p* < .001, Figure 3a, black). When absolute thresholding was applied, the ICC also increased with stronger thresholds, reaching a maximum of .76, again at the strongest threshold level (# streamlines removed = 40 streamlines, Figure 3b, black, Table S1). These results indicate that the edges retained after thresholding have more consistent weight distributions between scan and rescan.

Similar results were observed in controls, but with higher ICC scores (Figure S1).

#### 3.1.3 Similarity in hub score

The ICC degree also increased after thresholding, from .78 (unthresholded) to .83 when fixed‐density thresholding was used (density = .08, *p* < .001, Figure 3a, red, Table S1), whereas the ICC of betweenness centrality showed an unstable profile and did not increase significantly after thresholding (Figure 3a, green). Regarding absolute thresholding, the ICC degree also increased from .78 to a maximum of .83 (Figure 3b, red), and ICC‐betweenness centrality did not increase with stronger thresholds.

For controls, the effect of thresholding on hubs scores was analogous (see Figure S1), albeit with higher scores.

### 3.2 Interindividual variation and sensitivity to changes over time in patients

**FIGURE 4:**
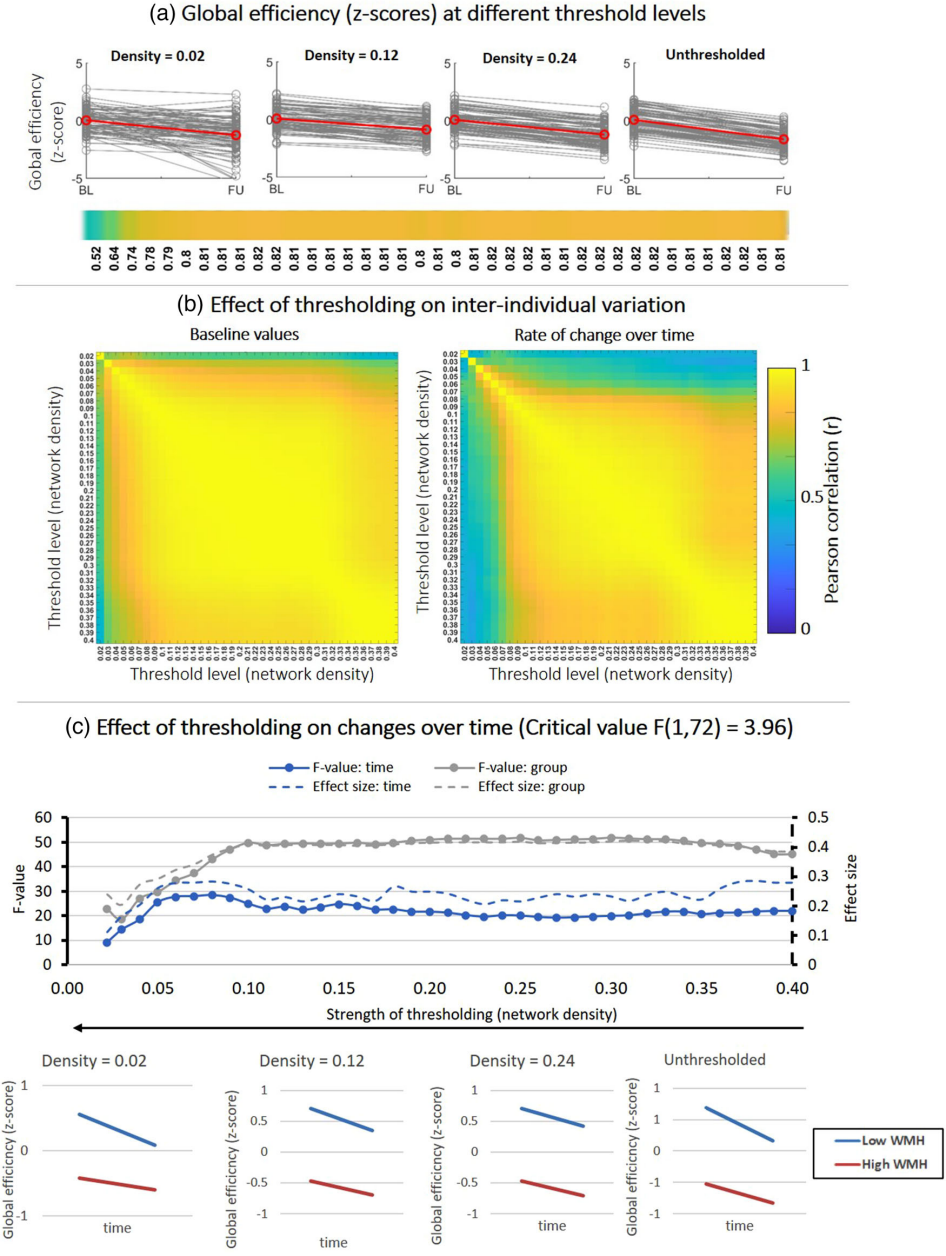
Effect of fixed‐density thresholding on global efficiency. (a) Spaghetti plots showing baseline and follow‐up z‐scores for different threshold levels. The red line represents the group average. The horizontal color bar shows correlations between baseline and follow‐up scores for each threshold level. (b) (Left) Correlation matrix containing Pearson correlations between baseline global efficiency scores of different threshold levels. High correlations indicate that the baseline scores are similar between threshold levels. (Right) Correlations between the rate of change over time obtained at different threshold levels. High correlations indicate that the individual rate of change is similar between threshold levels. (c) Impact of thresholding on the sensitivity to detect changes over time and group differences in patients stratified by WMH volume. (Top) F‐values and effect sizes were calculated using mixed ANOVA with time as within subject factor and group (low vs. high WMH) as between‐subject factor. Left axis corresponds to F‐values and right axis represents the effect sizes for each effect: time (blue), group (gray). (Bottom) Average change over time in global efficiency for patients with low vs. high WMH volume. Patients with high WMH have lower efficiency scores and both groups declined over time

**FIGURE 5:**
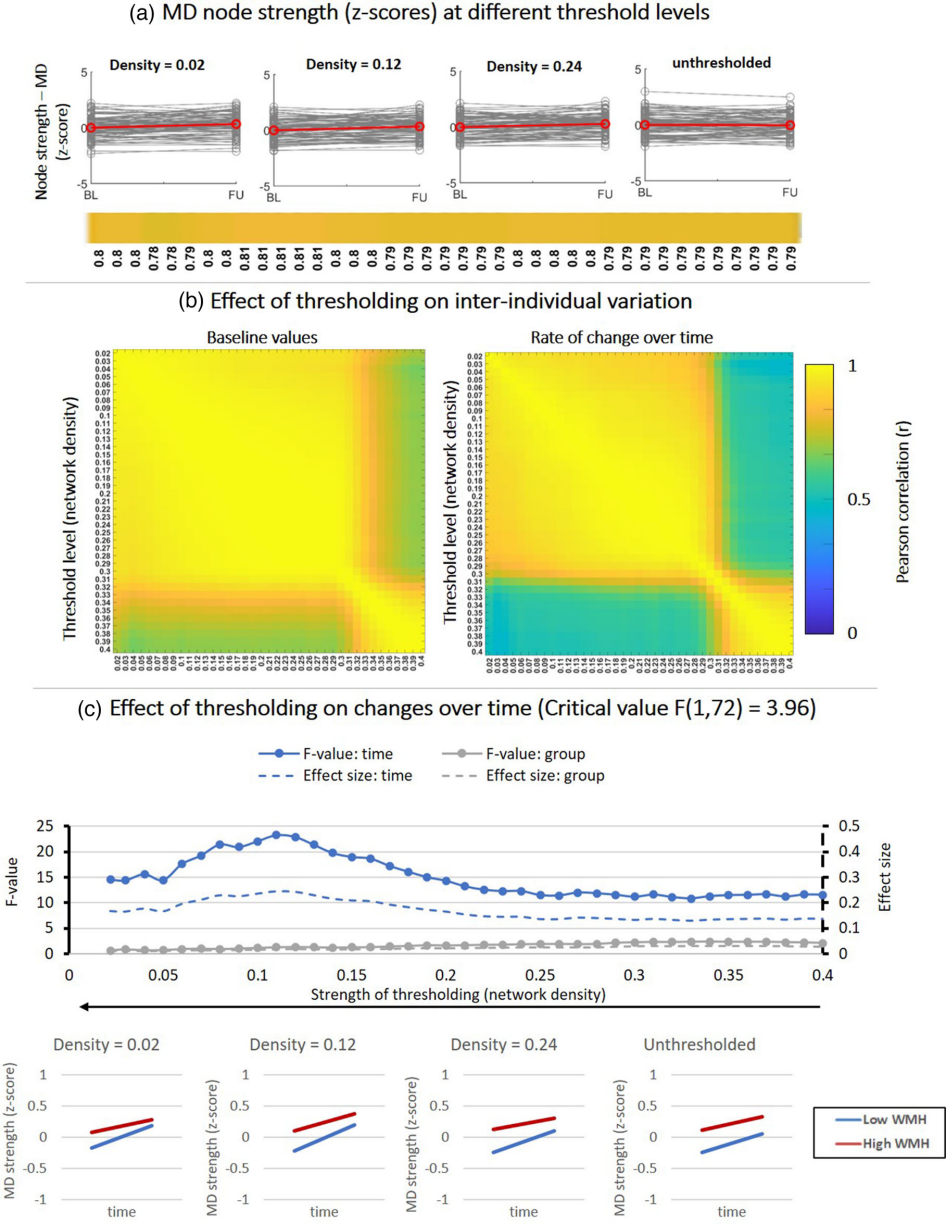
Effect of fixed‐density thresholding on MD‐weighted node strength. (a) Baseline and follow‐up z‐scores for different threshold levels. The horizontal color bar shows correlations between baseline and follow‐up scores within each threshold level. (b) (Left) Correlation matrix containing Pearson correlations coefficients between baseline scores of different threshold levels. (Right) Correlations between rate of change over time obtained at different threshold levels. (c) Impact of thresholding on the sensitivity to detect changes over time and group‐differences in patients stratified by WMH volume. (Top) F‐values and effect sizes for the effect s of time (blue), group (gray), interaction term (orange). (Bottom) Average change over time in MD‐weighted strength for patients with low vs. high WMH volume

**FIGURE 6:**
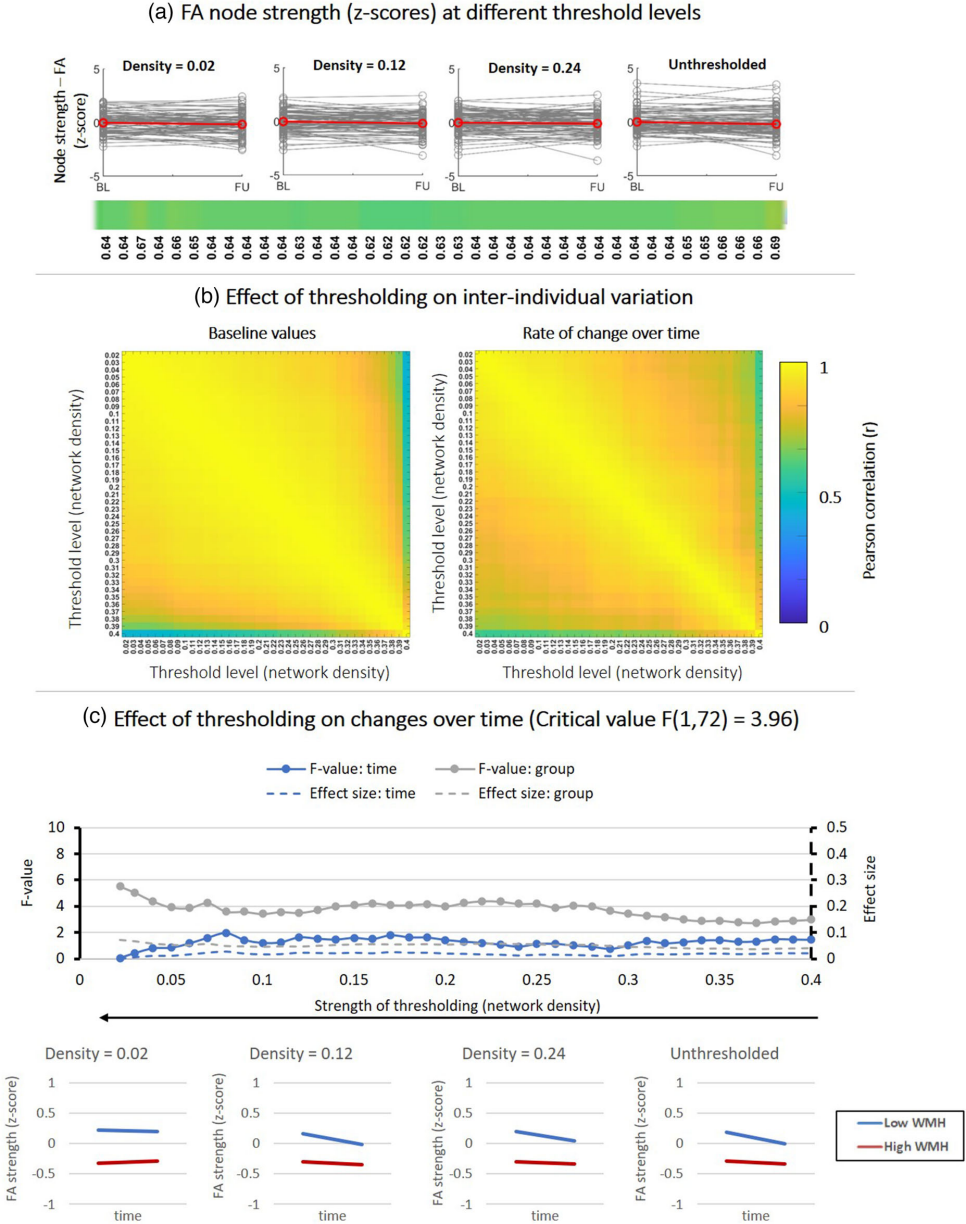
Effect of fixed‐density thresholding on FA‐weighted node strength. (a) Baseline and follow‐up z‐scores for different threshold levels. The horizontal color bar shows correlations between baseline and follow‐up scores within each threshold level. (b) (Left) Correlation matrix containing Pearson correlations coefficients between baseline scores of different threshold levels. (Right) Correlations between rate of change over time obtained at different threshold levels. (c) Impact of thresholding on the sensitivity to detect changes over time and group‐differences in patients stratified by WMH volume. (Top) F‐values and effect sizes for the effect s of time (blue), group (gray), interaction term (orange). (Bottom) Average change over time in FA‐weighted strength for patients with low vs. high WMH volume

The analyses assessing interindividual variation and sensitivity to changes in global efficiency, MD‐ and FA‐weighted node strength in patients are shown in Figures 4, 5, 6. In each figure, “panel a” contains spaghetti plots summarizing baseline and follow‐up z‐scores, as well as a correlations between baseline and follow‐up, within each threshold level. From a biological perspective, these correlations indicate whether patients maintain their relative position in group from baseline to follow‐up, which would then be reflected in parallel lines in the spaghetti plots, and strong correlations. “Panel b (left)” shows correlation matrices between baseline values of different threshold levels. These correlations indicate whether individual patients maintain their relative baseline scores across different thresholds. Threshold levels that do not disturb this interindividual variation of baseline scores should show high correlations. Likewise, “panel b (right)” shows a correlation matrix but for the rate of decline over time across thresholds, indicating whether individual patients maintain the same rate of decline between thresholds. “Panel c” depicts the sensitivity to detect time and group effects in patients stratified by WMH volume for different threshold levels. If a statistically significant change over time is found, this will be reflected in an F‐value (time) > critical value. The magnitude of this effect is given by the Cohen's d. Similarly, if there is significant difference between patients with low and high WMH volume, this group‐effect is given by the F‐value (group) and corresponding effect size.

Since absolute thresholding produced a large number of disconnected nodes, some brain regions got excluded from the analysis, not allowing us to always evaluate global efficiency over the same network of 90 nodes. It is well known that global network measures are highly dependent on the number of nodes (van Wijk et al., 2010). Therefore, for the analysis regarding the sensitivity to disease effects, we focused on the fixed‐density approach where the size of the network is maintained regardless of the threshold level, whereas the results for absolute thresholding are shown in the Supporting Information (Figures S1–S3).

#### 3.2.1 Global efficiency

Figure 4 shows results for global efficiency. In Figure 4a, the spaghetti plots indicate that before thresholding there is a clear variation in global efficiency between subjects, with an apparent decline from baseline to follow‐up. The lines of the subject initially run in parallel before thresholding, reflected in high a correlation between baseline and follow‐up scores (*r* = .81). After thresholding, this variation of baseline and follow‐up scores was maintained (*r* ≈ .8). The relation between baseline and follow‐up scores was only disturbed when networks were thresholded to densities < .05, implying that interindividual differences between subjects is no longer maintained.

In Figure 4b, we quantified whether the interindividual variation of subjects and the individual rate of change over time are maintained between different threshold levels. Regarding baseline values (left matrix), z‐scores of global efficiency remained robust between density thresholds of .40 and .05 (*r* > .9). In other words, the distance of each subject to the group mean at baseline was unchanged, as long as networks were not thresholded to densities below .05. The same was true for the change over time (right matrix). The individual rate of change from baseline to follow‐up was unaffected for densities between .40 and .10 (*r* > .9). Overall, these correlations analyses show that global efficiency scores of individual subjects do not change after thresholding (.10 < density < .40).

In Figure 4c, we statistically tested whether the apparent decline over time in global efficacy observed in the spaghetti plots was statistically significant, and whether patients with low WMH differed from high WMH volume. Before thresholding (density = .40), there was significant effect of time (blue curve), indicating a decline in global efficiency from baseline to follow‐up (F (1, 72) = 22; *p* < .01; Cohen's d = .29). This decline is in line with previous reports (Tuladhar et al., 2020). Regarding the group effect (gray curve), patients with larger WMH volumes had a significantly lower global efficiency (F (1, 72) = 45; *p* < .01; Cohen's d = .4). There was no interaction of time × group, with both groups of patients declining at the same rate. After thresholding, the sensitivity to detect time and group effects was robust, with F‐values and effect sizes remaining relatively constant for a wide range of densities between .40 and .10. This result indicates that these threshold levels preserve the sensitivity to effects of time and group observed originally before thresholding. The absolute thresholding method resulted in larger changes on the interindividual variation and sensitivity do detect time and group effects (Figure S1). Only a narrow range of thresholds preserved the sensitivity to detect time and group‐effects.

#### 3.2.2 MD‐weighted node strength

Figure 5 describes the same analysis as Figure 4, but for MD‐weighted node strength. By observing the spaghetti plots on Figure 5a, MD appears unchanged from baseline to follow‐up on a group level.

However, the correlation matrices on Figure 5b reveal that between a density of .4 and .30, MD scores remain initially unchanged (*r* > .9, note the block of high correlations on the bottom right of the matrix). When stronger thresholds were applied (i.e., when more noisy connections were removed, densities < .30), MD scores obtained at these threshold levels no longer resemble those obtained for thresholds between .40 and .30 but did not change further for the remaining thresholds (*r* > .9, for densities between .30 and .02).

In Figure 5c, we tested the sensitivity to detect changes over time. Before thresholding, there was a significant but small effect of time on MD (F (1, 72) = 12; *p* < .01; Cohen's d = .1). The group‐effect and the interaction time × group were not significant. After thresholding, the sensitivity to detect an effect of time was increased (i.e., larger F‐values) and highest for density thresholds around .12. Note that these were the threshold levels that also improved the consistency of network architecture over time. This indicates that removing noisy connections improves the sensitivity to detect small changes within individuals over time in local weight‐based metrics such as MD. Absolute thresholding produced larger changes on interindividual variation, but did not eliminate the sensitivity to effects of time and group (Figure S2).

#### 3.2.3 FA‐weighted node strength

Figure 6 shows the results for FA‐weighted node strength. Thresholding appears to have a small effect on the relation between baseline and follow‐up FA values as illustrated by the spaghetti plots and the correlations between baseline and follow‐up (Figure 6a).

The correlations matrices (Figure 6b) further show that the interindividual variation of baseline values (left matrix) remained robust for all threshold levels between .37 and .02 (*r* > .90). In other words, the position of each individual subject relative to the group mean did not change for thresholds between .37 and .02. The same result was obtained for the individual rate of change over time (right matrix).

Figure 6c shows that before thresholding no significant time and group effects were detected. Thresholding did not improve the sensitivity and effect of time, but did improve the sensitivity do detect a small effect of group for densities < .30: (F (1, 72) = 5; *p* < .05; Cohen's d = .05).

Again, absolute thresholding caused larger changes on interindividual variation but also improved the detection group‐effects after the first threshold levels (Figure S2).

## 4 DISCUSSION

In this work, we evaluated the impact of thresholding on scan–rescan brain networks of patients with SVD to assess how thresholds that improve scan–rescan network reproducibility in healthy young subjects affect (1) consistency in network architecture in these patients over a longer time period and (2) sensitivity to detect biological effects. Our results indicate that threshold levels that improve the reproducibility in controls also generate more consistent network architecture over time in patients. The similarity between scan and rescan for characteristics such as the location of edges detected, edge‐weights, and hub scores improved after thresholding. We also showed that the natural interindividual variation in outcome measures used to assess disease effect is preserved within threshold levels where the network architecture is consistent. Furthermore, the sensitivity to detect statistical group differences between patients with low vs. high WMH burden was maintained.

Preceding our work, several studies had examined the effect of thresholding on reproducibility of network architecture, including the binary topology, edges detected, edge‐weights, and graph metrics in healthy, mostly young, controls (Andreotti et al., 2014; Buchanan et al., 2014; Owen et al., 2013). As expected, our results with controls were in line with studies that explicitly showed that networks become more reproducible after thresholding (e.g., Buchanan et al., 2020; Messaritaki et al., 2019; Roine et al., 2019). In patients where disease effects are monitored over time, the rescan interval is typically much longer. Therefore, the same thresholding methods and threshold levels that are reported to improve reproducibility in controls might not directly apply. We used SVD as an exemplar condition, because network metrics have been shown to be relevant for this disease, but also because SVD‐related brain injury such as white matter hyperintensities, brain atrophy, and enlarged ventricles can impact the performance of tractography and network reconstruction. Current fiber tractography methods do not explicitly account for such factors, which can lead to erroneous estimations of white matter pathways and presence of more false positives in the network. Our results with patients show that the consistency of edges detected and their respective weights are improved when both thresholding methods are used, suggesting that the edges removed are noisy connections with a more random weight distribution (Messaritaki et al., 2019; Zalesky et al., 2016). Thresholding also improved the ICC of degree, meaning that if we were to define hubs nodes based on degree, a more consistent set of hubs would be detected between scans. Notably, the betweenness centrality was less consistent between scans, which could be explained by the fact that this metric depends not only on edges directly connected to a specific node but also on edges connected to distant nodes. Since the betweenness centrality quantifies the proportion shortest paths that go through given node, removing only one edge (which can be directly or indirectly connected to that node) can have a large impact on that shortest path. Thus, the betweenness centrality is more susceptible to disruptions when edges are removed (Drakesmith et al., 2015; Segarra & Ribeiro, 2014).

The two thresholding methods had distinct threshold levels to achieve optimal reproducibility. For the fixed‐density approach, similarity scores improved with decreasing density (i.e., stronger thresholds), before drastically decreasing for densities < .05. This reproducibility profile could be explained by the proportion of false positive at low densities and by the MST (Zalesky et al., 2016). The MST was incorporated to ensure that networks remain connected and avoid fragmented nodes. Since, by definition, the MST cannot contain connections that form cycles, a certain proportion of low‐weight connections (i.e., potential false positives) must be included in order to keep the network connected, meaning that at very low densities, the effect of these false positives is stronger. A potential disadvantage of thresholding networks to fixed‐density is that it can lead to confounding effects when comparing groups or datasets with different distributions of edge weights. In a group with higher edge weights, this would lead to ignoring potentially important edges with strong weights, while in a group with lower edge weights, this would lead to including weak or potentially spurious edges (van Wijk et al., 2010). Regarding the absolute thresholding approach, the similarity scores increased with mild thresholds (2–5 streamlines). Since this approach works by removing all connections with a weight below a certain number of streamlines, only the highest weighted connections survive when strong thresholds are used. A major downside of this method, also evident in our dataset, is that it quickly creates fragmented nodes, which means that some brain regions are no longer part of the network. Furthermore, differences in brain size or absolute number of streamlines computed for each subject results in largely different networks between subjects or over time. Thus, a “one size fits all” type of threshold is not ideal. For datasets of patients with similar characteristics as those included in this study, we therefore recommend using fixed‐density thresholds between .08 and .20 to achieve optimal network consistency while keeping all network nodes connected. This is also in line with previous research that estimated the density of structural connectomes to lie between .05 and .30 (Hagmann et al., 2008; Roberts et al., 2017). One of the main arguments against thresholding networks of patients in the attempt to improve network consistency over repeated scans is that this procedure could remove biological or disease‐related effects (Drakesmith et al., 2015; McColgan et al., 2018). This concern can be relevant in studies trying to identify disease effects at the level of subnetwork and/or individual connections, rather than pathological changes in large‐scale brain network topology (Petersen et al., 2020). In the SVD field, this type of analyses could help understanding how diffuse and/or focal damage in certain brain areas affects cognitive function. In those scenarios, applying thresholds could erroneously cut connections that are affected by pathology. On the other hand, for global network metrics, it is more desirable to assess disease effects on networks with a more consistent scan–rescan architecture, less affected by noisy connections that can confound the results. Detection of intraindividual changes over time in patients is generally one of the more challenging tasks in terms of sensitivity. Such changes may be detected at a group level, but the individual trajectories of patients tend to get overshadowed by noise if techniques are insensitive. Therefore, analysis of interindividual variation over time can be performed with more confidence if networks are voided of noisy connections. In the second part of our analysis, we show that the concerns about removal of disease effects by thresholding may not always be justified. For global efficiency, interindividual variation of subjects at baseline and their individual rate of change over time remained robust for fixed‐density thresholds > .10. Before thresholding, global efficiency was able to capture significant differences between patients with low and high WMH volume, which is in line with previous research where white matter lesion load was associated with lower global efficiency (Heinen et al., 2018). After fixed‐density thresholding, a wide‐range of densities (.40–.10) preserved the sensitivity to detect these disease‐related effects, suggesting that changes in global metrics can be consistently detected over multiple threshold levels (de Brito Robalo et al., 2020; Drakesmith et al., 2015). Absolute thresholding had a stronger impact interindividual variation and sensitivity to time and group effects (see Supporting Information), due to the fact that this thresholding approach creates disconnected nodes during, thereby changing the size of the network and disrupting global network metrics (van Wijk et al., 2010).

Regarding MD‐weighted node strength, the sensitivity to detect time effects was improved after thresholding (density ≈ .12). Since this network metric is based on edge weights, the measurements after thresholding are obtained over a smaller and more consistent set of connections, thereby decreasing the standard error and improving the sensitivity to detect small effect sizes. This hypothesis is also supported by previous work that examined associations between edge‐weights and age and showed that connections retained in the network after thresholding were significantly more associated with age than those removed (Buchanan et al., 2020). FA‐weighted node strength was lower in patients with higher WMH volume but did not significantly decline over time. These effects were not affected by thresholding.

Strengths of this work include the use of two distinct datasets, with different MRI protocols, different subject groups, and rescan intervals. In this manner, it was possible to directly test whether thresholds that improve the reproducibility in high‐quality scans with short rescan intervals also have the same effect on scans of patients, with longer follow‐up intervals. Our study also had some limitations. We analyzed the effects of thresholding only on network metrics that have shown association with disease effects in patients with SVD, such global efficiency and node strength. Thus, our results cannot be directly generalized to all network measures and all patient populations. Further investigation on disease effects reflected by other network metrics is required (e.g., local metrics or disease effects at the level of subnetworks). Our analysis was focused on the most popular weight‐based thresholding methods that directly remove connections from each individual network, without the need to create a group‐level network to determine a criterion to remove connections. In this manner, test–retest consistency of network architecture can be evaluated on an individual basis. Other scan–rescan studies have examined thresholding approaches that are not based on edge‐weights but rather on group‐level consistency and is less biased towards the length of streamlines (Buchanan et al., 2020; Roberts et al., 2017). Their findings also point that stringency of threshold can improve network consistency. However, future work should also analyze how consistency‐based thresholding improves group‐level consistency of network architecture (e.g., across scanners) in similar datasets and whether disease effects are preserved. Our results could also have been influenced by the choice of parcellation scheme (Zalesky et al., 2010), tractography algorithm (Bastiani et al., 2012), weighting scheme, among other factors. Thus, these results need to be tested using different network reconstruction pipelines.

## 5 CONCLUSION

Our study demonstrates the effects of weight‐based thresholding on longitudinal brain networks of patients with SVD. We showed that thresholding, particularly with fixed‐density approaches, can produce more consistent network architectures in patients scanned over longer time periods, while preserving disease‐related effects. Our work sheds a light on how to make informed decisions when applying thresholds in studies with a longitudinal design and how such choices can potentially influence the statistical significance of the results. A good practice for longitudinal studies that intend to apply weight‐based thresholds would be to first examine which threshold levels generate the most consistent network architecture over time and then verify if those threshold levels also preserve the interindividual variation in metrics that will be used as outcome for the study (e.g., global efficiency).

## CONFLICT OF INTEREST

The authors declare no conflict of interest.

## Supporting information

Supporting Information

## Data Availability

The data that support the findings of this study are available from the corresponding author upon request.
