# Supplementary material for: Impact of thresholding on the consistency and sensitivity of diffusion MRI‐based brain networks in patients with cerebral small vessel disease
Source: Brain Behav. 2022 Apr 12;12(5):e2523. doi: 10.1002/brb3.2523 (PMC9120729; doi:10.1002/brb3.2523)
Supplement: Supplementary file 1 — Supporting Information [file BRB3-12-e2523-s001.docx]

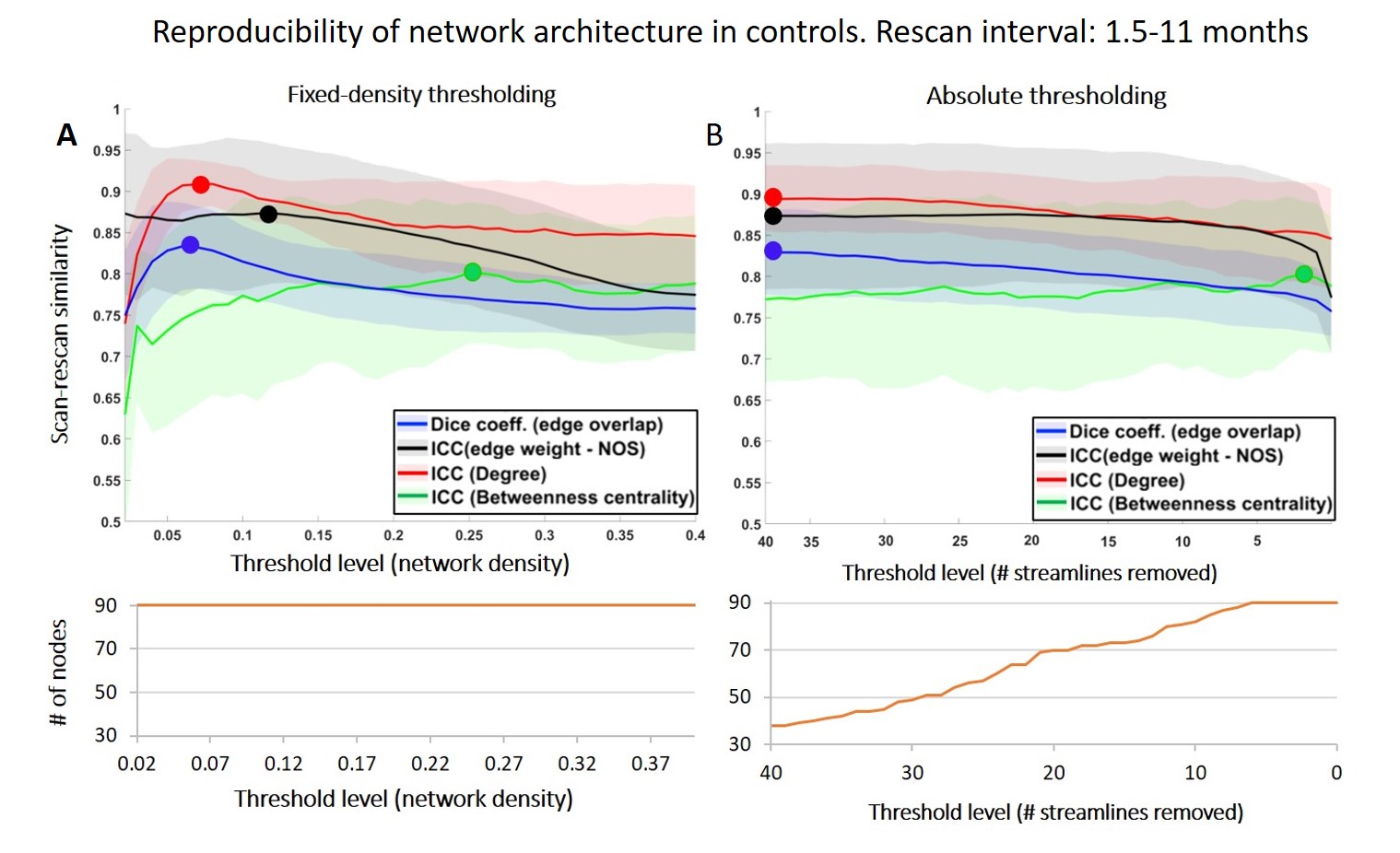


**Figure S1:** Reproducibility of network architecture between scan and rescan in controls (rescan interval = 1.5 – 11 months). In each plot, the x-axis represents the threshold level, with the strength of thresholding increasing from right to left. Note that for the fixed-density approach the stronger the threshold the lower the density, whereas for the absolute approach, the stronger the threshold, the higher the number of streamlines removed. The curve in orange under each plot shows the number of nodes that remain in the network after thresholding. The initial number of nodes for the unthresholded network was N = 90 nodes. Blue: dice similarity of edges, black: ICC of edge-weights; red: ICC of degree; green: ICC of betweenness centrality. The markers highlight maximum value in each curve, and the shaded areas represent the standard deviation.

Table 1: Similarity of network architecture between scan and rescan in patients, before and after thresholding

|  | Metric | Unthresholded | Thresholded | Effect size (Cohen’s d) | p-value |
| --- | --- | --- | --- | --- | --- |
| Fixed-density thresholding | Dice coefficient | 0.70 ± 0.04 | 0.76 ± 0.06 | 1.5 | <0.001 |
|  | ICC (edge weight – NOS) | 0.71 ± 0.06 | 0.75 ± 0.09 | 0.73 | <0.001 |
|  | ICC (degree) | 0.78 ± 0.10 | 0.83 ± 0.06 | 0.9 | <0.001 |
|  | ICC (betweenness centrality) | 0.70 ± 0.12 | 0.70 ± 0.12 | 0.12 | 0.75 |
| Absolute thresholding | Dice coefficient | 0.70 ± 0.04 | 0.76 ± 0.02 | 1.76 | <0.001 |
|  | ICC (edge weight – NOS) | 0.71 ± 0.06 | 0.76 ± 0.08 | 0.75 | <0.001 |
|  | ICC (degree) | 0.78 ± 0.10 | 0.83 ± 0.13 | 0.7 | <0.001 |
|  | ICC (betweenness centrality) | 0.70 ± 0.12 | 0.72 ± 0.2 | 0.19 | 0.06 |

Effect sizes and p-values obtained from paired samples t-test.

Table 2: Similarity of network architecture between scan and rescan in controls, before and after thresholding

|  | Metric | Unthresholded | Thresholded | Effect size (Cohen’s d) | p-value |
| --- | --- | --- | --- | --- | --- |
| Fixed-density thresholding | Dice coefficient | 0.76 ± 0.03 | 0.83 ± 0.07 | 2.1 | <0.001 |
|  | ICC (edge weight – NOS) | 0.77 ± 0.08 | 0.84 ± 0.10 | 1.5 | <0.001 |
|  | ICC (degree) | 0.85 ± 0.04 | 0.89 ± 0.07 | 0.81 | <0.001 |
|  | ICC (betweenness centrality) | 0.79 ± 0.10 | 0.80 ± 0.11 | 0.14 | 0.12 |
| Absolute thresholding | Dice coefficient | 0.76 ± 0.03 | 0.82 ± 0.05 | 1.79 | <0.001 |
|  | ICC (edge weight – NOS) | 0.77 ± 0.08 | 0.84 ± 0.12 | 1.2 | <0.001 |
|  | ICC (degree) | 0.85 ± 0.04 | 0.89 ± 0.06 | 0.77 | <0.001 |
|  | ICC (betweenness centrality) | 0.79 0.10 | 0.80 ± 0.16 | 0.1 | 0.34 |

Effect sizes and p-values obtained from paired samples t-test.

***Inter-individual variation and sensitivity to changes over time – absolute thresholding***


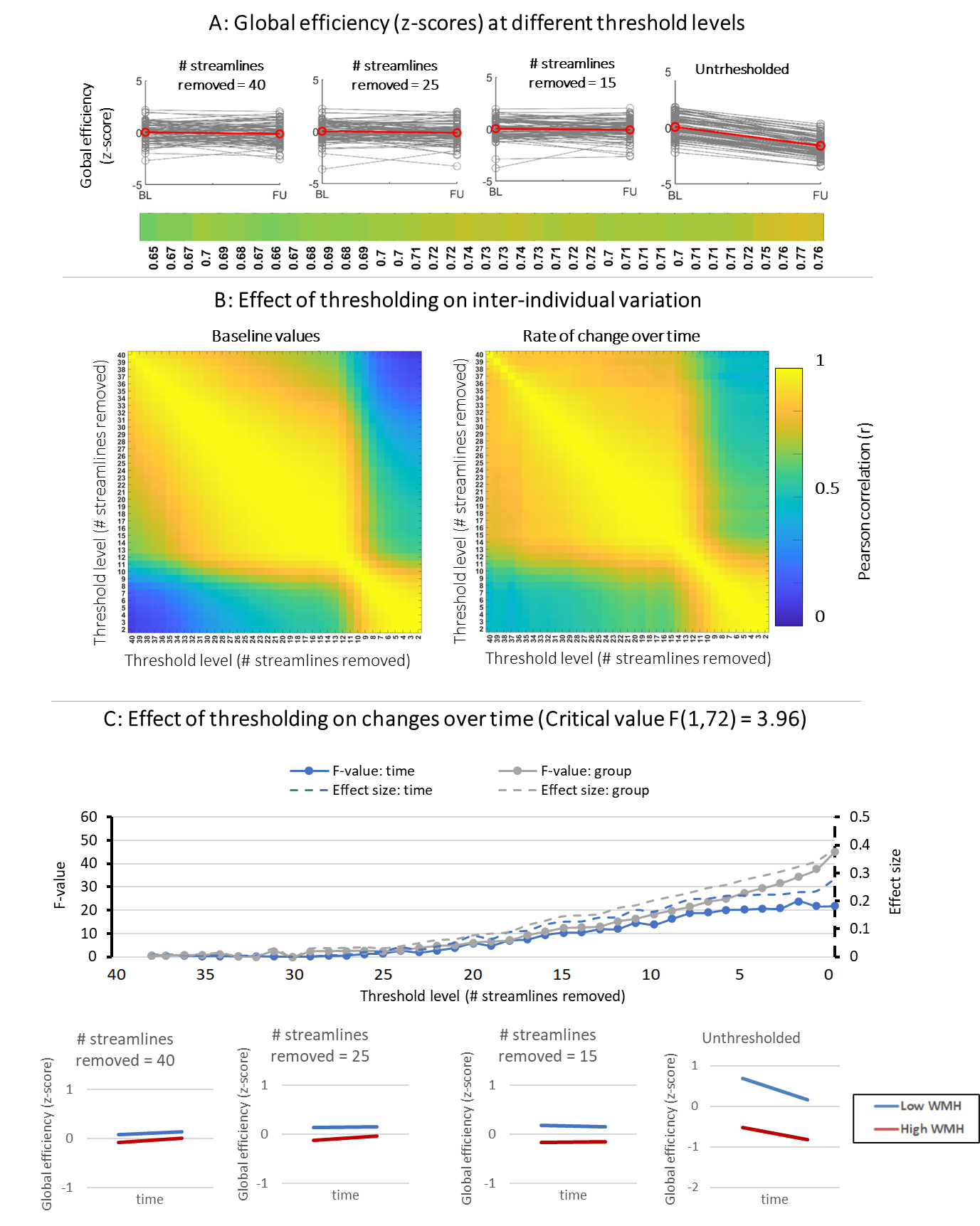


**Figure S1:** Effect of absolute thresholding on global efficiency. A: Spaghetti plots showing baseline and follow-up z-scores for different threshold levels. The red line represents the group average. The horizontal color bar shows correlations between baseline and follow-up scores for each threshold level. B: (Left) Correlation matrix containing Pearson correlations between baseline global efficiency scores of different threshold levels. High correlations indicate that the baseline scores are similar between threshold levels. (Right) Correlations between the rate of change over time obtained at different threshold levels. High correlations indicate that the individual rate of change is similar between threshold levels. C: Impact of thresholding on the sensitivity to detect changes over time and group-differences in patients stratified by WMH volume. (Top) F-values and effect sizes were calculated using mixed ANOVA with time as within subject factor and group (low vs. high WMH) as between-subject factor. Left axis corresponds to F-values and right axis represents the effect sizes for each effect: time (blue), group (gray). (Bottom) Average change over time in global efficiency for patients with low vs. high WMH volume. Patients with high WMH have lower efficiency scores and both groups declined over time.


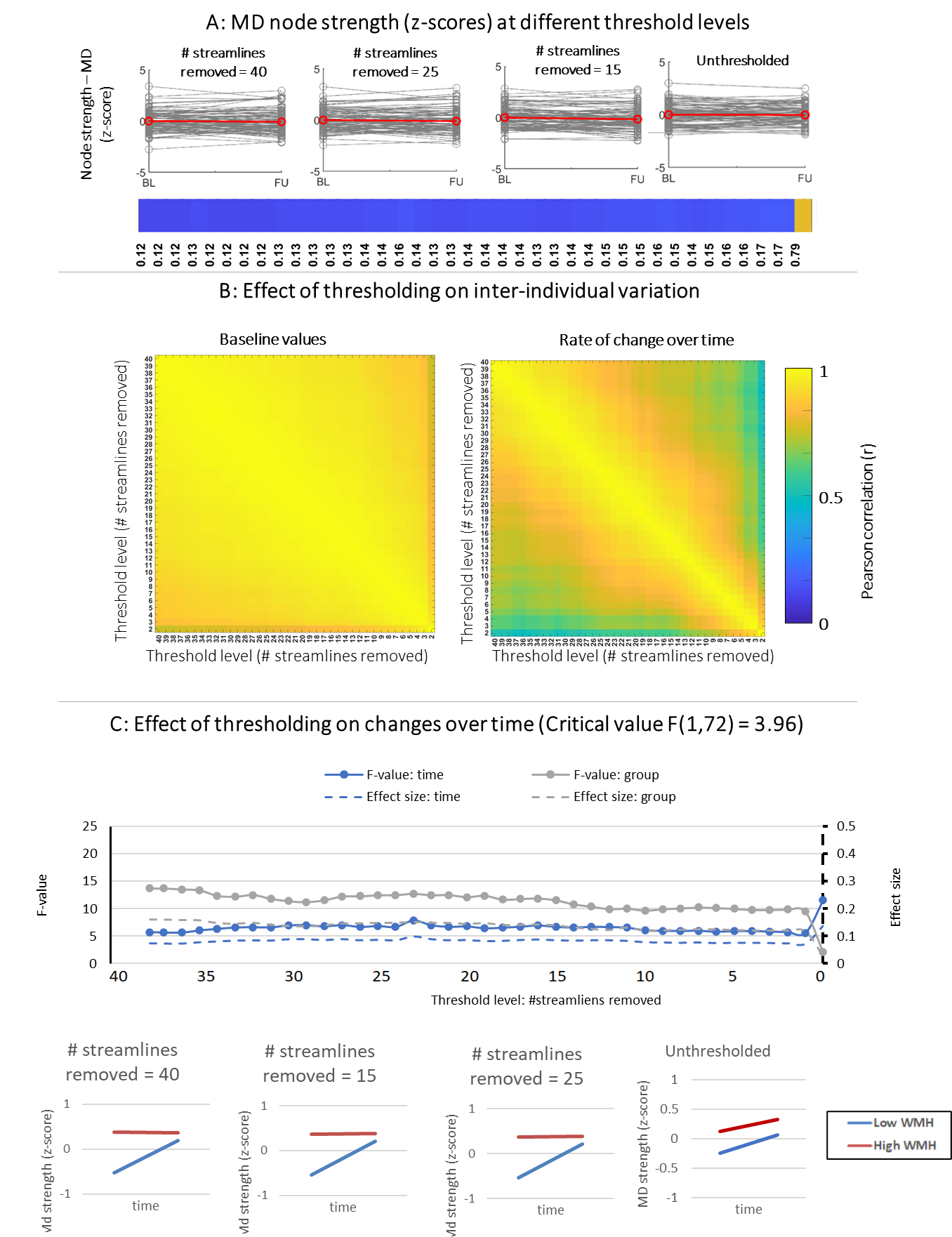


**Figure S2:** Effect of absolute thresholding on MD-weighted node strength. A: Baseline and follow-up z-scores for different threshold levels. B: (Left) Correlation matrix containing Pearson correlations coefficients between baseline scores of different threshold levels. (Right) Correlations between rate of change over time obtained at different threshold levels. C: Impact of thresholding on the sensitivity to detect changes over time and group-differences in patients stratified by WMH volume. (Top) F-values and effect sizes for the effect s of time (blue), group (gray), interaction term (orange). (Bottom) Average change over time in MD-weighted strength for patients with low vs. high WMH volume.


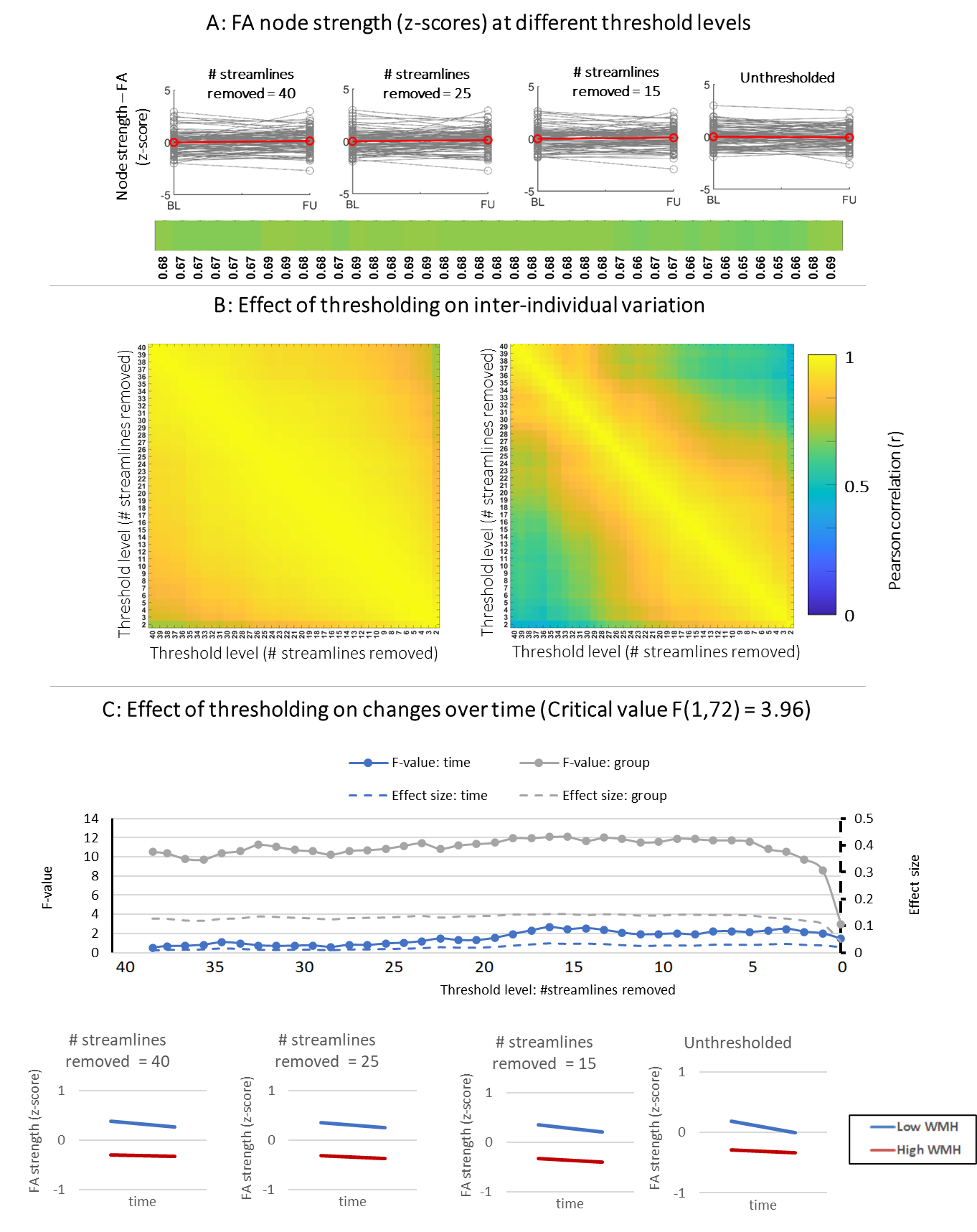


**Figure S3:** Effect of absolute thresholding on FA-weighted node strength. A: Baseline and follow-up z-scores for different threshold levels. B: (Left) Correlation matrix containing Pearson correlations coefficients between baseline scores of different threshold levels. (Right) Correlations between rate of change over time obtained at different threshold levels. C: Impact of thresholding on the sensitivity to detect changes over time and group-differences in patients stratified by WMH volume. (Top) F-values and effect sizes for the effect s of time (blue), group (gray), interaction term (orange). (Bottom) Average change over time in FA-weighted strength for patients with low vs. high WMH volume.
